# Support Vector Machine-Based Schizophrenia Classification Using Morphological Information from Amygdaloid and Hippocampal Subregions

**DOI:** 10.3390/brainsci10080562

**Published:** 2020-08-15

**Authors:** Yingying Guo, Jianfeng Qiu, Weizhao Lu

**Affiliations:** 1 Medical Engineering and Technology Research Center, Shandong First Medical University & Shandong Academy of Medical Sciences, Taian 271016, China; GuoY20000219@163.com (Y.G.); jfqiu100@gmail.com (J.Q.); 2 Department of Radiology, Shandong First Medical University & Shandong Academy of Medical Sciences, Taian 271016, China

**Keywords:** amygdala, hippocampus, machine learning

## Abstract

Structural changes in the hippocampus and amygdala have been demonstrated in schizophrenia patients. However, whether morphological information from these subcortical regions could be used by machine learning algorithms for schizophrenia classification were unknown. The aim of this study was to use volume of the amygdaloid and hippocampal subregions for schizophrenia classification. The dataset consisted of 57 patients with schizophrenia and 69 healthy controls. The volume of 26 hippocampal and 20 amygdaloid subregions were extracted from T1 structural MRI images. Sequential backward elimination (SBE) algorithm was used for feature selection, and a linear support vector machine (SVM) classifier was configured to explore the feasibility of hippocampal and amygdaloid subregions in the classification of schizophrenia. The proposed SBE-SVM model achieved a classification accuracy of 81.75% on 57 patients and 69 healthy controls, with a sensitivity of 84.21% and a specificity of 81.16%. AUC was 0.8241 (*p* < 0.001 tested with 1000-times permutation). The results demonstrated evidence of hippocampal and amygdaloid structural changes in schizophrenia patients, and also suggested that morphological features from the amygdaloid and hippocampal subregions could be used by machine learning algorithms for the classification of schizophrenia.

## 1. Introduction

Schizophrenia is a complicated mental disorder characterized by auditory hallucinations, paranoid or bizarre delusions, and disorganized speech and thinking [1]. Siblings and children of schizophrenia patients have a higher genetic risk of the disorder and are often accompanied with attentional lapses and memory impairments [2]. Although it is not completely clear what causes schizophrenia, it is believed that schizophrenia is related to genetic factor, environmental factors, and brain alterations [3].

Neuroimaging studies have revealed structural and functional alterations in schizophrenia brain as compared with healthy controls [4]. Among neuroimaging techniques, magnetic resonance imaging (MRI) is a widely used noninvasive technique, which provides structural information on cell loss and metabolic changes [5]. Compared with fMRI, structural MRI (sMRI) is less sensitive to noise and could be acquired with higher spatial resolution [6]. Previous studies have shown that sMRI has played an important role in the analysis of neurological diseases [7]. In terms of schizophrenia, sMRI studies have shown cortical and subcortical alterations, which are related to language impairments both in schizophrenia patients and genetic high-risk individuals for developing schizophrenia [8,9]. The volume of limbic system, including the hippocampus, amygdala, parahippocampal gyrus, etc., could be severed as indicators of schizophrenia as studies have shown subcortical structural changes in schizophrenia and high-risk individuals, and have attributed the structural changes to the impending development of schizophrenia [8,9]. Both the amygdala and hippocampus are important nuclei in the limbic system [8,10]. Okada et al. have demonstrated that volume of the bilateral amygdala and hippocampus of schizophrenia patients was smaller than that of healthy controls [11]. Zheng et al. have shown reduced volume in the left hippocampal tail and Cornu Ammonis 1 (CA1) in schizophrenia patients [10].

Current diagnosis of schizophrenia is based on observed behaviors and psychiatric symptoms [12]. However, in many cases, psychiatrists are divided on the diagnosis of schizophrenia due to lack of quantitative measures such as biomarkers [13]. Therefore, it is of clinical importance to find biomarkers to diagnose schizophrenia. Machine learning based on MRI measures, or the so-called multivariate pattern analysis technique, provides a promising way to find biomarkers for schizophrenia classification [12,13,14]. Xiao et al. created a support vector machine (SVM) model which could classify schizophrenia patients and normal subjects based on whole brain gray matter densities from sMRI [15]. Cao et al. achieved classification of schizophrenia patients with combined analysis of single nucleotide polymorphisms and fMRI data based on sparse representation [16]. Recent studies have reviewed MRI-based machine learning methods for classification of schizophrenia and have found that the performance of machine learning methods for schizophrenia classification varied from 0.54 to 0.95 in terms of area under the curve (AUC) [17,18]. The machine learning algorithms used for classification included SVM, neural network, k-nearest neighbors (KNN), random forest (RF), etc. [17,18].

The hippocampus and amygdala are important subcortical nuclei which have strong associations with the severity and progression of schizophrenia [9,11], but they have not been used by machine learning algorithms for schizophrenia classification. Therefore, the purpose of this study was to explore whether morphological information from the hippocampus and amygdala could be used for schizophrenia classification based on machine learning techniques.

## 2. Material and Methods

**Figure 1:**
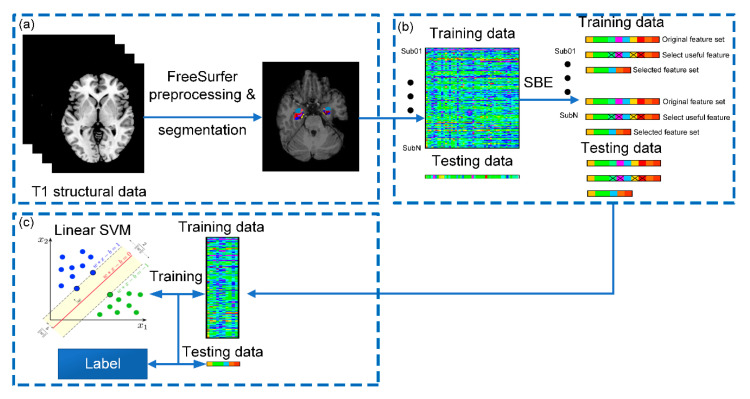
Schematic diagram of the proposed classification framework: (**a**) structural MRI (sMRI) preprocessing, hippocampal and amygdaloid subregion segmentation; (**b**) feature selection via sequential backward elimination (SBE) algorithm; and (**c**) classification using a linear support vector machine (SVM). Sub01~SubN represent all enrolled subjects.

Figure 1 demonstrates the schematic diagram of our proposed classification framework, consisting of image preprocessing, feature selection, and classification. We will give a detailed description for each step in this section.

### 2.1. Participants and sMRI Acquisition

sMRI data were acquired from the Center for Biomedical Research Excellence database (http://fcon_1000.project.nitrc.org/indi/retro/cobre.html). The dataset received full approval of local ethics committees in accordance with the Declaration of Helsinki. All subjects gave informed consent and their anonymity was preserved in the dataset. A total of 147 samples were obtained, including 72 patients with schizophrenia and 75 control subjects. The diagnosis of schizophrenia patients was based on the Structured Clinical Interview for the Diagnostic and Statistical Manual of Mental Disorders, 4th edition (DSM-IV SCID). The subjects were all right-handed. Specifically, 15 patients with schizophrenia and 6 healthy controls were excluded during data preprocessing, leaving a total of 126 participants, including 57 patients with schizophrenia and 69 healthy controls. Among the 57 schizophrenia patients, there were 3 disorganized type (295.1), 37 paranoid type (295.3), 9 residual type (295.6), 4 schizoaffective type (295.7), and 4 unspecified type (295.9).

T1-weighted MRI data were acquired using a Siemens Trio Tim 3T scanner (Munich, Germany). MPRAGE sequence was used with the following parameters: echo time (TE) = 1.64 ms, time of repetition (TR) = 2.530 ms, field of view (FOV) = 256 × 256 mm, flip angle = 7°, slice thickness = 1.0 mm, and voxel size = 1 × 1 × 1 mm^3^.

### 2.2. Imaging Processing

In this study, the reconstruction of cortical surface was carried out on T1-weighted images using FreeSurfer 6.0 [19]. Image preprocessing included the following steps: motion correction, brain extraction, Talairach transformation, intensity correction, and brain tissue segmentation [10]. After preprocessing, quality control was performed by a certified neuroradiologist, 15 patients with schizophrenia and 6 healthy controls were excluded because there were errors in skull stripping or segmentation.

**Figure 2:**
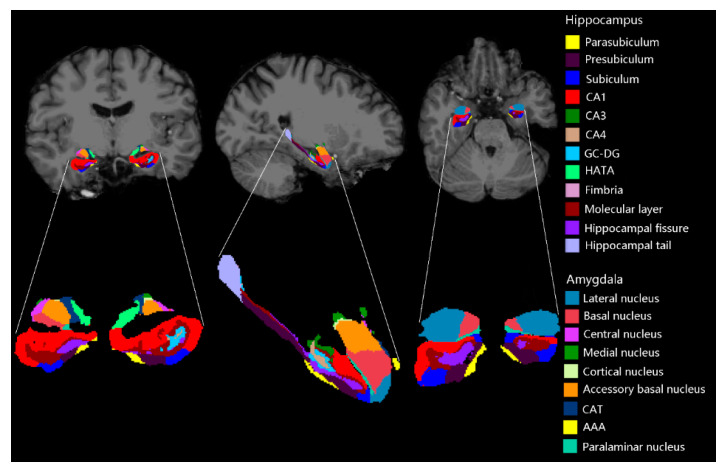
Subregions of the hippocampus and amygdala for one of the subjects.

The hippocampal and amygdaloid subregions were segmented using FreeSurfer development version [20] as demonstrated in Figure 2 [21,22]. We selected 46 structural features including 26 hippocampal features and 20 amygdaloid features. The 26 hippocampal features were: mean volume of the hippocampal tail, subiculum, CA1, CA3, CA4, hippocampal fissure, presubiculum, parasubiculum, molecular layer, granule cell layer of the dentate gyrus, fimbria, hippocampal-amygdala-transition-area (HATA), and whole hippocampus in the bilateral hemispheres. Optimally, 20 amygdaloid features included: mean volume of the lateral nucleus, basal nucleus, accessory basal nucleus, anterior amygdaloid area (AAA), central nucleus, medial nucleus, cortical nucleus, corticoamygdaloid-transition area (CAT), paralaminar nucleus, and whole amygdala in the bilateral hemispheres.

Then, the sMRI features were normalized to a range between 0 and 1 by linear scaling between the minimal and maximal values of each feature. A binary label with 1 for schizophrenia patients and −1 for healthy controls was used. GC-DG: granule cell layer of the dentate gyrus.

### 2.3. Feature Selection

In machine learning, some features are irrelevant for classification, so excluding certain features not only reduces computational complexity but also improves classification accuracy [14,15]. Sequential backward elimination (SBE) algorithm has been applied by several state-of-the-art studies and has achieved better results compared with other feature selection algorithms [23,24]. Therefore, in this study, SBE was adopted for feature selection, and classification error rate of a linear SVM was configured as the criterion function that SBE used to eliminate features and to determine when to terminate.

Starting from the full feature set, SBE created a candidate feature subset by sequentially eliminating each of the feature, which has not yet been eliminated in each backward step. Then, for each candidate feature subset (each backward step), SBE performed leave-one-out cross-validation by repeatedly calling the criterion function with different training set and test set. SBE used training set to train the linear SVM, then predicted values for test set using that model, and output classification error rate. If eliminating a certain feature reduced classification error rate, this feature would be eliminated, and vice versa. SBE algorithm repeated backward steps and stopped until there was no decrease in classification error rate.

We also adopted several competing feature selection approaches for the comparison with the proposed SBE algorithm.

#### 2.3.1. Sequential Selection and Its Variants

Three other sequential selection algorithms were adopted, i.e., sequential forward selection (SFS), sequential forward floating selection (SFFS), and sequential backward floating selection (SBFS).

Unlike SBE, SFS started from an empty set and created a candidate feature subset by sequentially selecting each feature, which has not yet been selected. The criterion function of SFS was the same with SBE.

SFFS also started with an empty set and created a candidate feature subset by sequentially selecting each feature in each forward step. Different from SFS algorithm, SFFS performed backward steps as long as the criterion function increased. SBFS started with the full feature set and performed each backward step by sequentially eliminating features from the full set. SBFS performed forward steps as long as the criterion function increased. In the current study, the criterion functions of SFFS and SBFS were the same as SBE.

#### 2.3.2. *T*-Test

The differentiation degree of each feature between schizophrenia patients and healthy controls was compared via two-sample *t*-test to test whether there were significant differences in the mean value of the features between the two groups. In this study, we used a threshold of |*t*| > 1.04 (*p* < 0.3) to exclude features and improve classification performance. The threshold value was determined by grid search.

#### 2.3.3. F-Score

F-score is a simple and effective metric for evaluation of a binary classification model [25]. Given the number of schizophrenia patients (n_+_) and healthy controls (n_−_), the F-score of the *i*th feature is defined as follows [25]:

(1) $$F\left(i\right)=\frac{{\left({\bar{x}}_{i}^{\left(+\right)}-{\bar{x}}_{i}\right)}^{2}+{\left({\bar{x}}_{i}^{\left(-\right)}-{\bar{x}}_{i}\right)}^{2}}{\frac{1}{n_{+}-1}\sum_{k=1}^{n_{+}}{\left(x_{k,i}^{\left(+\right)}-{\bar{x}}_{i}^{\left(+\right)}\right)}^{2}+\frac{1}{n\_-1}\sum_{k=1}^{n\_}{\left(x_{k,i}^{\left(-\right)}-{\bar{x}}_{i}^{\left(-\right)}\right)}^{2}}$$

where ${\bar{x}}_{i}$, ${\bar{x}}_{i}^{\left(+\right)}$, and ${\bar{x}}_{i}^{\left(-\right)}$ are the mean values of the ith feature of the whole, positive, and negative data sets, respectively; $x_{k,i}^{\left(+\right)}$ and $x_{k,i}^{\left(-\right)}$ are the ith feature of the kth positive and negative instance, respectively. In our study, the threshold was set at F-score < 0.009 determined by grid search.

#### 2.3.4. Random Forest

Gini Impurity Index from the RF model is often used to evaluate the importance of features in machine learning [26]. In the current study, we borrowed this metric for feature selection. We kept the feature whose Gini Impurity Index was not equal to 0.

There were only 46 sMRI features in this study, so in order to evaluate the effect of feature selection, we also used the full feature set to train the linear SVM classifier.

### 2.4. SVM and Evaluating Metrics

#### 2.4.1. Linear SVM

SVM is a type of supervised learning method, which is widely used in machine learning field [15]. In this study, LIBSVM toolbox based on the MATLAB platform was used to implement SVM classifier [27,28]. Linear SVM has been widely applied in multivariate pattern analysis due to its high accuracy, generalization, and interpretability [15,17,18]. Therefore, in this study, linear kernel was selected. For the hyperparameter C in the SVM classifier, which controls the balance between classification error and model generalization, was set at 112 after a coarse grid search to obtain the best performance. A weight of 1.3 was added to the schizophrenia group, making the parameter C for the patients’ class to 1.3×C to maintain a balance between the two classes. In order to obtain a reliable performance and to avoid overfitting, leave-one-out cross-validation strategy was used in our study. In each cross-validation fold, 125 subjects were used for training and the remaining one subject was selected to test the model. The iteration continued for 126 times. To represent relative contribution of different features for schizophrenia classification, we accumulated the absolute value of the weight across all cross-validation folds.

#### 2.4.2. Competing Algorithms

In this study, three competing algorithms were used to classify schizophrenia patients, namely, KNN, RF, and feedforward neural network (FNN).

A KNN model was configured with Euclidean distance as distance measure. The value of k was set to 5. In the RF model, 100 trees were used. An FNN with three layers was configured with 46 neurons in the input layer, 15 neurons in the hidden layer, and 1 neuron in the output layer. tansig function was defined as the activation function for the hidden and output layer. Mean-square error was defined as cost function and Levenberg–Marquardt algorithm was used as weight update algorithm for the FNN. All three classifiers were implemented using build-in functions of MATLAB.

#### 2.4.3. Evaluating Metrics

Accuracy, sensitivity, and specificity were used to evaluate the performance of the classifiers. The three metrics were computed in each cross-validation fold and were finally averaged to get the mean values. In addition, receiver operating characteristic (ROC) analysis was also used to evaluate the performance of the classifiers. Area under the curve (AUC) calculated from the ROC curve was used as an indicator of classification performance. Permutation test was applied to explore whether the AUC obtained through the proposed model was significantly higher than AUC of a random guess by randomly permuting the labels of the training data 1000 times prior to the training step and then followed by the entire classification process. The AUCs were obtained across all permutations and the *p* value was calculated as the proportion of AUCs that were equal to or greater than the AUC obtained by the proposed methods. Statistical significance was set at *p* < 0.05.

### 2.5. Post Hoc Analysis

According to the cumulative absolute weight, eight features with top cumulative absolute weights were selected by the linear SVM classifier. General linear model was applied to explore whether the features were discriminative between schizophrenia patients and healthy controls with age and gender as nuisance covariates. False discovery rate (FDR) correction was used to control false positives, and *p* < 0.05 was considered statistically significant. Pearson correlation analysis was performed to explore the relationship between demographic information and the eight features in schizophrenia group. FDR correction with *p* < 0.05 was considered statistically significant. Furthermore, independent *t*-test was used to explore whether the features were discriminative between paranoid type and other types of schizophrenia.

## 3. Results

### 3.1. Demographic Information

Table 1 illustrates the demographic information for the 57 schizophrenia patients and 69 healthy controls. The differences in age and gender between the two groups were assessed by independent *t*-test and chi-square test, respectively. There were no significant differences in age and gender between schizophrenia patients and healthy controls. 

### 3.2. Performance of the Classifier

Table 2 shows the results of different feature selection approaches. It was obvious that SBE approach associated with a linear SVM classifier was superior to other feature selection approaches in terms of accuracy, sensitivity, specificity, AUC, and *p* value obtained from permutation test. Other feature selection approaches could also improve classification accuracy by 2–20% compared with the full feature set, which indicated that the step of feature selection in our study is useful for improving classification performance. However, it was also worth mentioning that the several approaches, namely, F-score, *t*-test, Gini Index, and SBFS decreased the specificity compared with the full feature set. In addition, it could be observed that number of features selected by SBE algorithm was more than that of others except for SBFS.

**Figure 3:**
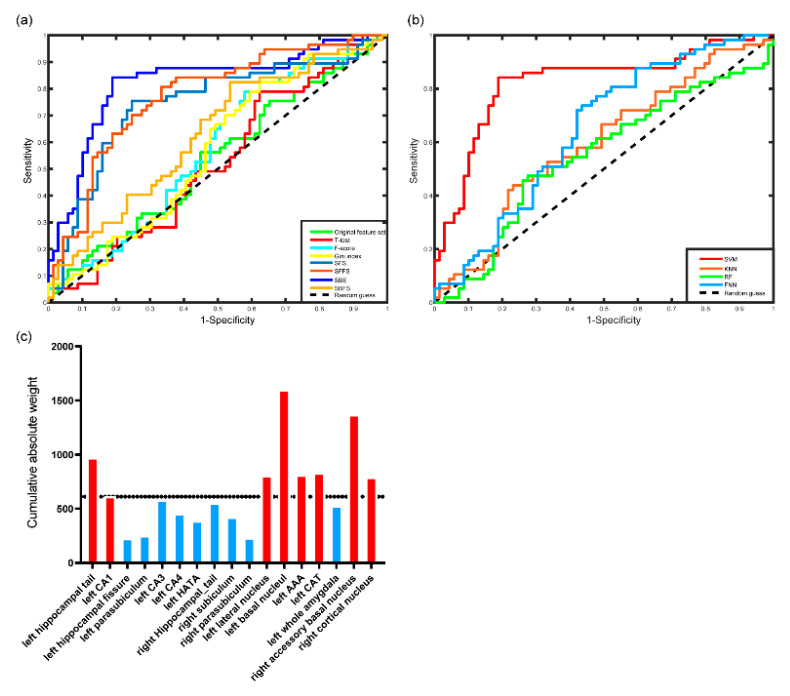
Results of SVM classification: (**a**) receiver operating characteristic (ROC) curves of the linear SVM classifier with the original feature set and 7 different feature selection approaches, (**b**) ROC curves of 4 classifiers, and (**c**) the features selected by sequential backward elimination (SBE) approach and their cumulative absolute weights. The regions with red color represent the 8 regions having top cumulative absolute weights.

The ROC curves of different feature selection approaches associated with linear SVM classifiers are shown in Figure 3a. SBE-SVM outperformed the full feature set and the other competing feature selection approaches associated with SVM classifiers.

Table 3 and Figure 3b show classification results of different classifiers. The linear SVM classifier outperformed competing classification algorithms in terms of accuracy, sensitivity, specificity, and AUC.

As shown in Table 2, we retained 17 features through SBE and ranked them according to their cumulative absolute weights. The regions with greater cumulative absolute weight were the left hippocampal tail and left CA1 of hippocampus, the left lateral nucleus, left basal nucleus, left AAA, left CAT, right accessory basal nucleus, and right cortical nucleus of amygdala, as shown in Figure 3c.

### 3.3. Post Hoc Analysis Results

**Figure 4:**
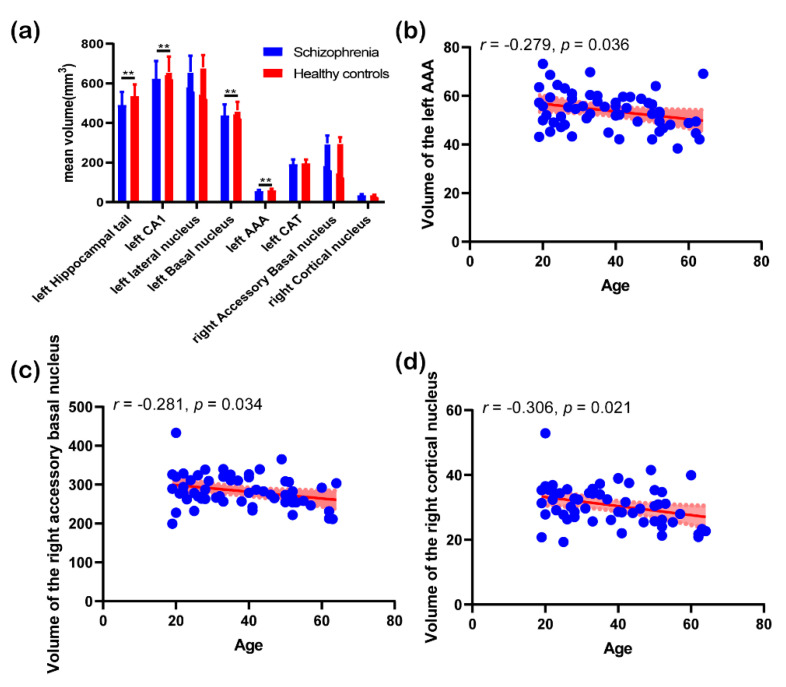
Results of post hoc analysis: (**a**) histogram of features with top cumulative absolute weight between the two groups. **, *p* < 0.05 (false discovery rate (FDR) corrected). (**b**) correlation analysis between mean volume of the left anterior amygdaloid area (AAA) and age; (**c**) correlation analysis between mean volume of the right accessory basal nucleus and age; and (**d**) correlation analysis between mean volume of the right cortical nucleus and age, the red area represents 95% confidence interval.

**Figure 5:**
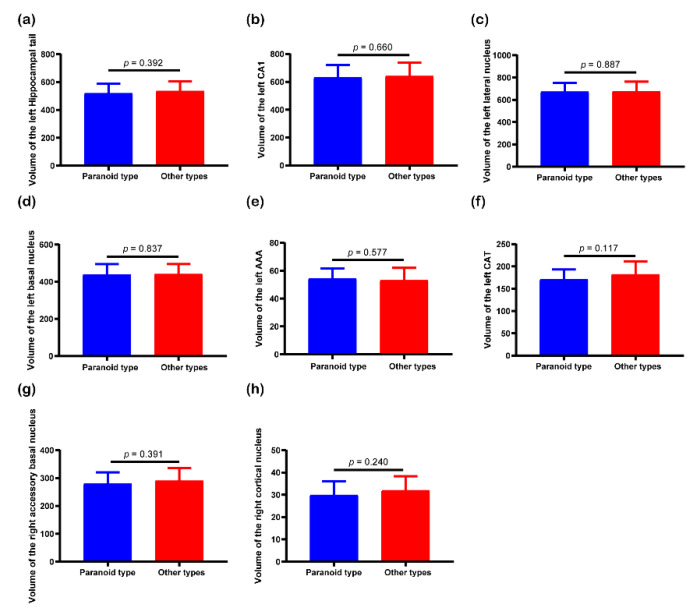
*T*-test results of mean volume of the (**a**) left hippocampal tail, (**b**) left Cornu Ammonis 1 (CA1), (**c**) left lateral nucleus, (**d**) left basal nucleus, (**e**) left AAA, (**f**) left corticoamygdaloid-transition area (CAT), (**g**) right accessory basal nucleus, and (**h**) right cortical nucleus between paranoid type and other types of schizophrenia.

Figure 4 displays the results of post hoc analysis. Post hoc results showed that there were significantly statistical differences in the volume of the left hippocampal tail (*p* = 0.004, FDR corrected), left CA1 (*p* = 0.04, FDR corrected), left basal nucleus (*p* = 0.011, FDR corrected), and left AAA (*p* = 0.004, FDR corrected) between schizophrenia patients and healthy controls. However, the volume of the other 4 hippocampal and amygdaloid subregions did not show significant differences between the two groups. In terms of correlation analysis, mean volume of the left AAA, right accessory basal nucleus, and right cortical nucleus showed negative correlations with age in schizophrenia group. Figure 5 shows the results of independent *t*-test. As shown in the figure, none of the 8 features were significantly discriminative between paranoid type and other types of schizophrenia.

## 4. Discussion

Diagnosis of schizophrenia is clinically dependent on psychiatric examinations since biomarkers that could accurately classify schizophrenia remain unknown [15,29]. Machine learning algorithms associated with neuroimaging features provide a promising way for schizophrenia diagnosis [18]. To date, machine learning algorithms including SVM, RF, KNN, FNN, and deep learning algorithms associated with fMRI and sMRI features have been used in schizophrenia diagnosis [17,18]. The performance of machine learning algorithms varied from 70% to 90% in terms of accuracy and from 0.54 to 0.95 in terms of AUC [17]. The high performance demonstrated that machine learning algorithms were useful in recognizing and detecting schizophrenia patients at an early stage.

In addition to schizophrenia classification, machine learning algorithms were useful for identifying biomarkers in schizophrenia. Indeed, previous studies have reported several brain regions with potential diagnostic values including the occipital and frontal gyrus [15,16,29]. Raymond et al. have used gray matter, white matter features, and cortical thickness to distinguish schizophrenia patients from healthy subjects, and they achieved a maximum accuracy of 77% [30]. Castellani et al. have chosen the dorsolateral prefrontal cortex as feature for machine learning, and have achieved a maximum accuracy of up to 84.09% [31]. In the current study, we focused on only two key subcortical nuclei, the amygdala and hippocampus and explored whether the features extracted from these two subcortical nuclei could be used by machine learning algorithm to classify schizophrenia. The SVM classifier based on morphological features from the amygdala and hippocampus had a relatively high accuracy in schizophrenia diagnosis compared with previous studies [17,18]. In addition, SVM has identified several amygdaloid and hippocampal subregions having potential values for schizophrenia classification. The results demonstrated that the proposed approach could be used in assisting clinical schizophrenia diagnosis and also revealed that the hippocampus and amygdala were closely involved in the pathology of schizophrenia, which was consist with previous studies [8,9,10,11,32].

Among the subregions with top contributions to the classification, group analysis showed that there were differences in the volume of the left hippocampal tail, left CA1, left basal nucleus, and left AAA between the two groups, which indicated that these features were discriminative between schizophrenia patients and healthy subjects. Zheng et al. have demonstrated volumetric decline of several subregions in the hippocampus and amygdala in schizophrenia and have speculated key role of these regions in the pathophysiology of schizophrenia [10]. In line with the previous study [10], our results might not only indicate the importance of these three features in the pathophysiology of schizophrenia but also prove the effectiveness of feature selection and cumulative absolute weight in schizophrenia classification.

There was evidence that the activation of postsynaptic 5-HT (1A) receptors in the hippocampal tail was related to stress adaptation and could prevent learned helplessness [33]. The decreased volume in the hippocampal tail indicated that schizophrenia patients might experience decreased learning and memory ability [10]. A study by Kesner et al. showed that CA1 could code the time sequence of events and CA1 was associated with intermediate-term memory [34]. In addition, Schobel et al. found that the CA1 in the hippocampal subregion was differentially targeted by schizophrenia and related psychotic disorders [35]. Several studies attributed differential changes in the CA1 as basal hypermetabolic activity, and demonstrated that dysfunction in the CA1 was a selective defect that predicted progression of schizophrenia [35,36]. Therefore, the contribution of CA1 in classification of schizophrenia might indicate that the left CA1 is closely related to the pathophysiology of schizophrenia.

In our study, we found that the lateral nucleus and CAT in the left amygdala could be used to classify schizophrenia. A previous study speculated that the left amygdala was more closely related to emotion processing in schizophrenia [10]. The lateral nucleus is the largest nucleus, and it is the first structure to appear in the anterior portion of the amygdala [10]. The lateral nucleus could be divided into dorsal, ventral intermediate, and ventral subdivisions [37]. Previous findings showed that the lateral nucleus was an important sensory interface which send projections to the CAT (a zone of confluence of the medial parvicellular basal nucleus, paralaminar nucleus, and sulcal periamygdaloid cortex) [38]. Several studies have demonstrated decreased volume in the lateral nucleus of amygdala in schizophrenia patients compared with normal controls and have concluded that reduced volume of the lateral nucleus could play an important role in the pathological process of schizophrenia [10,39]. The CAT is located in the medial border of the amygdala with connections to many regions such as the hippocampus and temporal cortex [37,40]. The CAT is also a zone of confluence in the amygdalohippocampal area [37,38]. Brown et al. demonstrated volumetric association of the bilateral CAT with neurological disorders such as major depressive disorder [41]. The contributions of the left lateral nucleus and left CAT in the classification of schizophrenia suggested that the two subregions might play more important roles in the pathophysiology of schizophrenia.

The present study showed that the accessory basal nucleus and cortical nucleus in the right amygdala could be used to classify schizophrenia. A neuroimaging study has found that volume of all subregions in the right amygdala was decreased in schizophrenia when compared with psychotic bipolar disorder [42]. The basal nucleus and accessory basal nucleus are the main input sources to the ventral striatum [43]. The ventral striatum and amygdala are two mesolimbic structures associated with psychosis in schizophrenia [44]. Therefore, the results may reflect a potential role of the accessory basal nucleus in psychosis in schizophrenia. The cortical nucleus is a small circular nucleus, which borders the accessory basal nucleus [10]. In addition, in a study with elderly schizophrenia patients and control group, Prestia et al. found that outer soft tissue of the cortical nucleus was lost in schizophrenia patients [45].

Correlation analysis demonstrated negative correlations between volume of the left AAA, right accessory basal nucleus and right cortical nucleus, and age in schizophrenia group. Although there was no significant difference in age between schizophrenia patients and healthy subjects in the present study, previous longitudinal studies have demonstrated that schizophrenia patients have significantly smaller volume in several subcortical nuclei compared with controls at baseline, and the volumetric changes of these subcortical nuclei with age in schizophrenia patients have similar trajectories compared with those of healthy controls [46,47]. According to previous studies [46,47], the contributions of these three subregions in schizophrenia classification may be more related to the pathophysiology of schizophrenia, rather than ageing. Nevertheless, the negative correlations could be explained by ageing-related neurodegeneration.

None of the eight subregions showed volumetric differences among different subtypes of schizophrenia, indicating that no neurobiological differences in the hippocampal and amygdaloid subregions exist among different subtypes of schizophrenia. In a recent study, Lutz et al. have found no large neurobiological differences between paranoid and nonparanoid schizophrenia [48]. In line with the previous study, our results indicated that subtypes of schizophrenia characterized by clinical phenomenology might have difficulty in resolving neurobiological heterogeneity in schizophrenia due to overlapping symptomatology and longitudinal instability [48], while machine learning may stand a chance of investigating neurobiological heterogeneity in schizophrenia [49].

There were several limitations that need to be addressed. First, this study lacked clinical information such as positive and negative syndrome scale (PANSS), which limited clinical interpretation of machine learning results. Future studies will focus on a larger study sample with completed clinical information. Second, we only separated the dataset into training set and test set due to relatively small study sample, the generalization of the proposed approach should be further validated using a larger study sample. Third, the proposed approach was only used to separate schizophrenia patients from healthy controls, however, other disorders with structural changes in the amygdala and hippocampus were not included in the current study.

## 5. Conclusions

In this study, we used the volume of the hippocampal and amygdaloid subregions extracted from sMRI data as features and used the SBE-SVM for the classification of schizophrenia. The classifier outperformed competing algorithms with a classification accuracy of 81.75%, and identified several hippocampal and amygdaloid subregions that had potential diagnostic value for schizophrenia. The proposed algorithm showed potential for assisting clinical diagnosis of schizophrenia. Neurobiological mechanism of the hippocampal and amygdaloid subregions in schizophrenia needs further investigation.

## Figures and Tables

**Table 1:** The demographic information of schizophrenia patients and healthy controls.

Index	Patients (*n* = 57)	Controls (*n* = 69)	T/χ2	*p*
Age	37.85 ± 13.63	35.32 ± 11.17	1.15 ^1^	0.252
Gender (F/M)	9/48	20/49	3.068 ^2^	0.08

^1^ Age was assessed by independent *t*-test. ^2^ Gender was assessed by chi square test.

**Table 2:** Evaluation metrics of seven feature selection approaches and the full feature set with a linear support vector machine (SVM) classifier.

Method	Accuracy	Sensitivity	Specificity	AUC	Feature number	*p*
Full feature set	53.97%	56.14%	55.07%	0.5362	46	0.226
F score	55.56%	70.17%	47.83%	0.5751	11	0.062
*T*-test	56.35%	78.95%	37.68%	0.5238	11	0.329
Gini Index	57.14%	64.91%	52.17%	0.5700	13	0.088
SFS	74.60%	75.44%	75.36%	0.7442	5	<0.001
SFFS	71.43%	80.70%	66.67%	0.7732	6	<0.001
SBFS	58.73%	82.46%	46.38%	0.6293	30	0.007
SBE	81.75%	84.21%	81.15%	0.8241	17	<0.001

**Table 3:** Evaluation metrics of four different classifiers.

Classifier	Accuracy	Sensitivity	Specificity	AUC
SVM	81.75%	84.21%	81.15%	0.8241
KNN	61.90%	52.63%	66.67%	0.5960
RF	58.73%	47.37%	72.46%	0.5489
FNN	57.14%	63.16%	59.42%	0.5934

## References

[1] Krishnan R.R., Keefe R., Kraus M. (2009). Schizophrenia is a disorder of higher order hierarchical processing. Med. Hypotheses.

[2] Seidman L.J., Giuliano A.J., Smith C.W., Stone W.S., Glatt S.J., Meyer E., Faraone S.V., Tsuang M.T., Cornblatt B. (2005). Neuropsychological functioning in adolescents and young adults at genetic risk for schizophrenia and affective psychoses: Results from the harvard and hillside adolescent high risk studies. Schizophr. Bull..

[3] Guloksuz S., Pries L., Delespaul P., Kenis G., Luykx J.J., Lin B.D., Richards A.L., Akdede B., Binbay T., Altınyazar V. (2019). Examining the independent and joint effects of molecular genetic liability and environmental exposures in schizophrenia: Results from the EUGEI study. World Psychiatry.

[4] Saarinen A.I.L., Huhtaniska S., Pudas J., Björnholm L., Jukuri T., Tohka J., Granö N., Barnett J.H., Kiviniemi V., Veijola J. (2020). Structural and functional alterations in the brain gray matter among first-degree relatives of schizophrenia patients: A multimodal meta-analysis of fMRI and VBM studies. Schizophr. Res..

[5] Andjus P.R., Danijela B., Vanhoutte G., Mitrecic D., Pizzolante F., Djogo N., Nicaise C., Kengne F.G., Gangitano C., Michetti F. (2009). In vivo morphological changes in animal models of amyotrophic lateral sclerosis and alzheimer’s-like disease: MRI Approach. Anat. Rec..

[6] Zou L., Zheng J., Miao C., McKeown M.J., Wang Z.J. (2017). 3D CNN based automatic diagnosis of attention deficit hyperactivity disorder using functional and structural MRI. IEEE Access..

[7] Tong T., Wolz R., Gao Q., Guerrero R., Hajnal J.V., Rueckert D. (2014). Multiple instance learning for classification of dementia in brain MRI. Med. Image Anal..

[8] Li X., Black M., Xia S., Zhan C., Bertisch H.C., Branch C.A., DeLisi L.E. (2015). Subcortical structure alterations impact language processing in individuals with schizophrenia and those at high genetic risk. Schizophr. Res..

[9] Satizabal C.L., Adams H.H., Hibar D.P., White C.C., Knol M.J., Stein J.L., Scholz M., Sargurupremraj M., Jahanshad N., Roshchupkin G.V. (2019). Genetic architecture of subcortical brain structures in 38,851 individuals. Nat. Genet..

[10] Zheng F., Li C., Zhang N., Cui D., Wang Z., Qiu J. (2019). Study on the sub-regions volume of hippocampus and amygdala in schizophrenia. Quant. Imaging Med. Surg..

[11] Okada N., Fukunaga M., Yamashita F., Koshiyama D., Yamamori H., Ohi K., Yasuda Y., Fujimoto M., Yahata N., Nemoto K. (2016). Abnormal asymmetries in subcortical brain volume in schizophrenia. Mol. Psychiatry.

[12] Hallak J., De Paula A., Chaves C., Bressan R.A., Machado-De-Sousa J. (2015). An overview on the search for schizophrenia biomarkers. CNS Neurol. Disord. Drug Targets.

[13] Razafsha M., Khaku A., Azari H., Alawieh A., Behforuzi H., Fadlallah B., Kobeissy F., Wang K.K., Gold M.S. (2015). Biomarker identification in psychiatric disorders. J. Psychiatr. Pr..

[14] Chyzhyk D., Savio A., Graña M. (2015). Computer aided diagnosis of schizophrenia on resting state fMRI data by ensembles of ELM. Neural Networks.

[15] Xiao Y., Yan Z., Zhao Y., Tao B., Sun H., Li F., Yao L., Zhang W., Chandan S., Liu J. (2017). Support vector machine-based classification of first episode drug-naïve schizophrenia patients and healthy controls using structural MRI. Schizophr. Res..

[16] Cao H., Duan J., Lin D., Shugart Y.Y., Calhoun V., Wang Y.-P. (2014). Sparse representation based biomarker selection for schizophrenia with integrated analysis of fMRI and SNPs. NeuroImage.

[17] Bracher-Smith M., Crawford K., Escott-Price V. Machine learning for genetic prediction of psychiatric disorders: A systematic review. Mol. Psychiatry.

[18] Steardo L., Carbone E.A., De Filippis R., Pisanu C., Segura-Garcia C., Squassina A., De Fazio P., Steardo L. (2020). Application of support vector machine on fMRI data as biomarkers in schizophrenia diagnosis: A systematic review. Front. Psychol..

[19] FreeSurfer 6.0. http://surfer.nmr.mgh.harvard.edu/.

[20] FreeSurfer development version. ftp://surfer.nmr.mgh.harvard.edu/pub/dist/freesurfer/dev.

[21] Iglesias J.E., Augustinack J.C., Nguyen K., Player C.M., Player A., Wright M., Roy N., Frosch M.P., McKee A.C., Wald L.L. (2015). A computational atlas of the hippocampal formation using ex vivo, ultra-high resolution MRI: Application to adaptive segmentation of in vivo MRI. NeuroImage.

[22] Saygin Z., Kliemann D., Iglesias J.E., Van Der Kouwe A., Boyd E., Reuter M., Stevens A., Van Leemput K., McKee A., Frosch M. (2017). High-resolution magnetic resonance imaging reveals nuclei of the human amygdala: Manual segmentation to automatic atlas. NeuroImage.

[23] Koutsouleris N., Kambeitz-Ilankovic L., Ruhrmann S., Rosen M., Ruef A., Dwyer D.B., Paolini M., Chisholm K., Kambeitz J., Haidl T. (2018). Prediction models of functional outcomes for individuals in the clinical high-risk state for psychosis or with recent-onset depression: A multimodal, multisite machine learning analysis. JAMA Psychiatry.

[24] Deng L., Zhang Q.C., Chen Z., Meng Y., Guan J., Zhou S. (2014). PredHS: A web server for predicting protein–Protein interaction hot spots by using structural neighborhood properties. Nucleic Acids Res..

[25] Liu F., Guo W., Fouche J.-P., Wang Y., Wang W., Ding J., Zeng L., Qiu C., Gong Q., Zhang W. (2013). Multivariate classification of social anxiety disorder using whole brain functional connectivity. Brain Struct. Funct..

[26] Svetnik V., Liaw A., Tong C., Culberson J.C., Sheridan R.P., Feuston B.P. (2003). Random forest: A Classification and regression tool for compound classification and QSAR modeling. J. Chem. Inf. Comput. Sci..

[27] Chang C.C., Lin C.J. (2011). LIBSVM: A library for support vector machines. ACM Trans. Intel. Syst. Tec..

[28] LIBSVM. https://www.csie.ntu.edu.tw/~cjlin/libsvm/.

[29] De Filippis R., Carbone E.A., Gaetano R., Bruni A., Pugliese V., Garcia C.S., De Fazio P. (2019). Machine learning techniques in a structural and functional MRI diagnostic approach in schizophrenia: A systematic review. Neuropsychiatr. Dis. Treat..

[30] Salvador R., Radua J., Canales-Rodríguez E.J., Solanes A., Sarró S., Goikolea J.M., Valiente-Gómez A., Monté G.C., Natividad M.D.C., Guerrero-Pedraza A. (2017). Evaluation of machine learning algorithms and structural features for optimal MRI-based diagnostic prediction in psychosis. PLoS ONE.

[31] Castellani U., Rossato E., Murino V., Bellani M., Rambaldelli G., Perlini C., Tomelleri L., Tansella M., Brambilla P. (2011). Classification of schizophrenia using feature-based morphometry. J. Neural Transm..

[32] Rajarethinam R., Dequardo J.R., Miedler J., Arndt S., Kirbat R., Brunberg J.A., Tandon R. (2001). Hippocampus and amygdala in schizophrenia: Assessment of the relationship of neuroanatomy to psychopathology. Psychiatry Res. Neuroimaging.

[33] Joca S.R.L., Padovan C.M., Guimaraes F.S. (2003). Activation of post-synaptic 5-HT(1A) receptors in the dorsal hippocampus prevents learned helplessness development. Brain Res..

[34] Kesner R.P., Lee I., Gilbert P. (2004). A behavioral assessment of hippocampal function based on a subregional analysis. Rev. Neurosci..

[35] Schobel S.A., Lewandowski N.M., Corcoran C., Moore H., Brown T., Malaspina D., Small S.A. (2009). Differential targeting of the CA1 subfield of the hippocampal formation by schizophrenia and related psychotic disorders. Arch. Gen. Psychiatry.

[36] Eastwood S., Falkai P., Bogerts B., Harrison P. (1995). Poly(A)+mRNA as a marker of metabolic activity in schizophrenia: Differential changes in parahippocampal gyrus and CA. Schizophr. Res..

[37] Fudge J.L., Decampo D.M., Becoats K.T. (2012). Revisiting the hippocampal–amygdala pathway in primates: Association with immature-appearing neurons. Neuroscience.

[38] Fudge J.L., Tucker T. (2009). Amygdala projections to central amygdaloid nucleus subdivisions and transition zones in the primate. Neuroscience.

[39] Pawel K., Helmut H., Valentina M., Woltersdorf F., Masson T., Ulfig N., Schmidt-Kastner R., Korr H., Steinbusch H.W.M., Hof P.R. (2007). Volume, neuron density and total neuron number in five subcortical regions in schizophrenia. Brain.

[40] Romanski L.M., LeDoux J.E. (1993). Information cascade from primary auditory cortex to the amygdala: Corticocortical and corticoamygdaloid projections of temporal cortex in the rat. Cereb. Cortex.

[41] Brown S., Rutland J.W., Verma G., Feldman R.E., Alper J., Schneider M., Delman B.N., Murrough J.M., Balchandani P. (2019). Structural MRI at 7T reveals amygdala nuclei and hippocampal subfield volumetric association with Major Depressive Disorder symptom severity. Sci. Rep..

[42] McMahon F., Lee D.S., Trinh H., Tward D., Miller M.I., Younes L., Barta P.E., Ratnanather J.T. (2015). Morphometry of the amygdala in schizophrenia and psychotic bipolar disorder. Schizophr. Res..

[43] Decampo D.M., Fudge J.L. (2013). Amygdala projections to the lateral bed nucleus of the stria terminalis in the macaque: Comparison with ventral striatal afferents. J. Comp. Neurol..

[44] Epstein J., Stern E., Silbersweig D. (1999). Mesolimbic activity associated with psychosis in schizophrenia: Symptom-specific PET studies. Ann. N. Y. Acad. Sci..

[45] Prestia A., Boccardi M., Galluzzi S., Cavedoa E., Adorniab A., Soricellic A., Bonettid M., Geroldiab C., Giannakopoulosef P., Thompson P. (2011). Hippocampal and amygdalar volume changes in elderly patients with Alzheimer’s disease and schizophrenia. Psychiatry Res..

[46] Van Haren N.E.M., Schnack H.G., Koevoets M.G., Cahn W., Pol H.H., Kahn R.S., Information P.E.K.F.C. (2016). Trajectories of subcortical volume change in schizophrenia: A 5-year follow-up. Schizophr. Res..

[47] Barth C., Jørgensen K.N., Wortinger L.A., Nerland S., Jönsson E.G., Agartz I. (2020). Trajectories of brain volume change over 13 years in chronic schizophrenia. Schizophr. Res..

[48] Lutz O., Lizano P., Mothi S.S., Zeng V., Hegde R.R., Hoang D.T., Henson P., Brady R., Tamminga C.A., Pearlson G. (2020). Do neurobiological differences exist between paranoid and non-paranoid schizophrenia? Findings from the bipolar schizophrenia network on intermediate phenotypes study. Schizophr. Res..

[49] Chand G.B., Dwyer D.B., Erus G., Sotiras A., Varol E., Srinivasan D., Doshi J., Pomponio R., Pigoni A., Dazzan P. (2020). Two distinct neuroanatomical subtypes of schizophrenia revealed using machine learning. Brain.

